# Ablation of MCL1 expression by virally induced microRNA-29 reverses chemoresistance in human osteosarcomas

**DOI:** 10.1038/srep28953

**Published:** 2016-06-30

**Authors:** Shuhei Osaki, Hiroshi Tazawa, Joe Hasei, Yasuaki Yamakawa, Toshinori Omori, Kazuhisa Sugiu, Tadashi Komatsubara, Tomohiro Fujiwara, Tsuyoshi Sasaki, Toshiyuki Kunisada, Aki Yoshida, Yasuo Urata, Shunsuke Kagawa, Toshifumi Ozaki, Toshiyoshi Fujiwara

**Affiliations:** 1 Department of Orthopaedic Surgery, Okayama University, Graduate School of Medicine, Dentistry and Pharmaceutical Sciences, Okayama 700-8558, Japan; 2 Department of Gastroenterological Surgery, Okayama University, Graduate School of Medicine, Dentistry and Pharmaceutical Sciences, Okayama 700-8558, Japan; 3 Center for Innovative Clinical Medicine, Okayama University Hospital, Okayama 700-8558, Japan; 4 Department of Medical Materials for Musculoskeletal Reconstruction, Okayama University Graduate School of Medicine, Dentistry and Pharmaceutical Sciences, Okayama 700-8558, Japan; 5 Oncolys BioPharma, Inc., Tokyo 105-0001, Japan

## Abstract

Osteosarcoma is a rare disease diagnosed as malignant bone tumor. It is generally refractory to chemotherapy, which contributes to its poor prognosis. The reversal of chemoresistance is a major clinical challenge to improve the prognostic outcome of osteosarcoma patients. We developed a tumor-specific replication-competent oncolytic adenovirus, OBP-301 (telomelysin) and assessed its synergistic effects with chemotherapeutic agents (cisplatin and doxorubicin) using human osteosarcoma cell lines and a xenograft tumor model. The molecular mechanism underlying the chemosensitizing effect of OBP-301 was evaluated in aspects of apoptosis induction. OBP-301 inhibits anti-apoptotic myeloid cell leukemia 1 (MCL1) expression, which in turn leads to chemosensitization in human osteosarcoma cells. The siRNA-mediated knockdown of MCL1 expression sensitized human osteosarcoma cells to common chemotherapeutic agents. We also found that upregulation of microRNA-29 targeting MCL1 via virally induced transcriptional factor E2F-1 activation was critical for the enhancement of chemotherapy-induced apoptosis in osteosarcoma cells. Telomerase-specific oncolytic adenovirus synergistically suppressed the viability of human osteosarcoma cells in combination with chemotherapeutic agents. The combination treatment also significantly inhibited tumor growth, as compared to monotherapy, in an osteosarcoma xenograft tumor model. Our data suggest that replicative virus-mediated tumor-specific MCL1 ablation may be a promising strategy to attenuate chemoresistance in osteosarcoma patients.

Osteosarcoma is a rare disease with less than 1,000 new cases every year diagnosed as malignant primary bone tumors in children and adolescents in the United States^1^. Despite recent advances in multi-agent chemotherapy and aggressive surgical resection, the poor response to chemotherapy is a major critical prognostic factor in osteosarcoma patients^2^,^3^. Chemotherapy-refractory osteosarcoma patients frequently show tumor recurrence, distant metastasis and poor prognosis. Increasing the chemotherapy dose induced short-lasting remission, but did not increase survival. The survival rate has remained unchanged over the past 30 years^2^. Therefore, the enhancement of chemosensitivity is a potential approach to improve the clinical outcome of osteosarcoma patients.

The molecular mechanism underlying the resistance to chemotherapy in osteosarcoma patients is poorly understood. One possible mechanism is the resistance to apoptosis induced by chemotherapeutic agents^4^,^5^. The B-cell lymphoma 2 (BCL2) family proteins are suspected to regulate apoptotic cell death caused by chemotherapeutic agents in human osteosarcoma cells^2^. The anti-apoptotic BCL2 family proteins, including BCL2 6, myeloid cell leukemia 1 (MCL1)^7^, and B-cell lymphoma-X large (BCL-XL)^8^, are frequently overexpressed in human sarcoma cells. Indeed, the suppression of BCL2^9^, MCL1^7^, and BCL-XL^8^ can enhance the chemosensitivity of human sarcoma cells. These findings suggest that anti-apoptotic BCL2 family proteins are potential therapeutic targets to improve the chemoresistance in osteosarcoma patients. Hence, the development of a novel therapy that efficiently suppresses the expression of anti-apoptotic BCL2 family proteins is needed.

Virus infection and replication produce exogenous viral proteins, many of which manipulate the host cellular machinery to allow viral persistence in the life-cycle. Indeed, adenoviral E1A, a gene product in the adenoviral early region, exerts tumor suppressive functions, including enhancement of chemotherapy-induced apoptosis via stabilization of tumor suppressors such as p53 and p21^10^ and inhibition of cell proliferation via suppression of epidermal growth factor receptor (EGFR)^11^ and HER2^12^. Adenoviral E1B55kDa protein also induces the proteolytic degradation of the Mre11-Rad50-NBS1 (MRN) complex, leading to the profound radiosensitization of human cancer cells^13^,^14^. Oncolytic virotherapy is a promising antitumor strategy to induce tumor-specific cell death^15^. These findings suggest that oncolytic viruses may influence the sensitivity of human osteosarcoma cells to chemotherapeutic agents.

In the present study, we show that genetically engineered telomerase-specific oncolytic adenovirus OBP-301 (telomelysin) efficiently kills human osteosarcoma cells and markedly sensitizes them to common chemotherapeutic agents. Notably, targeting the anti-apoptotic BCL2 family protein MCL1 via OBP-301-induced microRNA-29 activation is critical as the underlying mechanism of the OBP-301-mediated chemosensitizing effect.

## Results

### *In vitro* cytotoxic effect of chemotherapeutic agents and OBP-301 in human osteosarcoma cells

**Figure 1:**
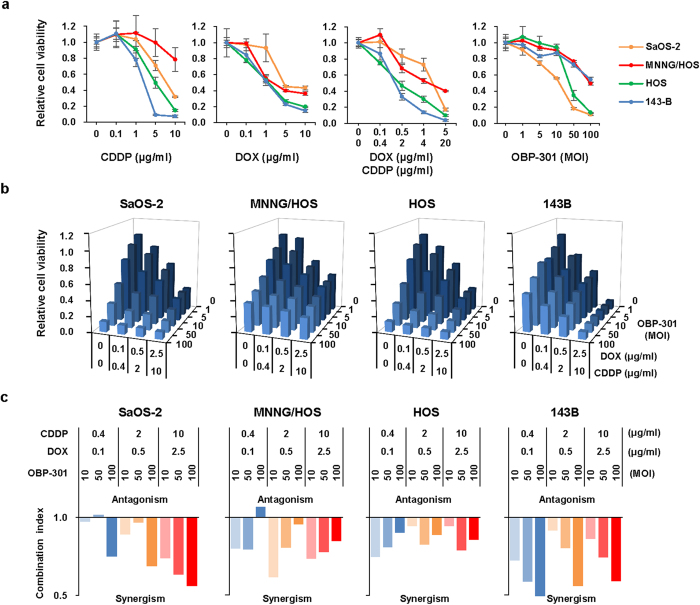
OBP-301 synergistically enhances the cytotoxic effect of chemotherapeutic agents in human osteosarcoma cells. (**a**) Four human osteosarcoma cell lines (SaOS-2, MNNG/HOS, HOS, and 143B) were treated with cisplatin (CDDP) or doxorubicin (DOX) at the indicated doses for 24 hours. In combined chemotherapy, cells were treated with CDDP and DOX at the clinically used ratio (CDDP:DOX = 4:1) for 24 hours. Cells were infected with OBP-301 at the indicated MOIs for 3 days. Cell viability was quantified using the XTT assay. Cell viability was calculated relative to that of the mock-infected group, which was set at 1.0. Cell viability data are expressed as mean values ± SD (n = 5). **(b)** In combination therapy with chemotherapy and OBP-301, cells were infected with OBP-301 at the indicated MOIs. Two days after viral infection, cells were further treated with CDDP and DOX (CDDP:DOX = 4:1) for 24 hours. **(c)** The combination index was calculated with the CalcuSyn software (BioSoft, Inc.). Synergism and antagonism were defined as interaction indices of <1 or >1, respectively.

We have developed a telomerase-specific replication-competent oncolytic adenovirus, OBP-301 (telomelysin), which induces tumor-specific cell death in a variety of human cancer cells^16^,^17^. To evaluate the therapeutic potential of chemotherapeutic agents and OBP-301 in human osteosarcoma cells, we first analyzed the *in vitro* cytotoxic effect of two chemotherapeutic agents, cisplatin (CDDP) and doxorubicin (DOX), which are frequently used for the treatment of osteosarcoma, and OBP-301 in 4 human osteosarcoma cell lines (MNNG/HOS, SaOS-2, HOS, and 143B). MNNG/HOS and SaOS-2 cells were relatively less sensitive to CDDP or DOX as compared to HOS and 143B cells (Fig. 1a). In combination with chemotherapeutic agents at the clinically used ratio (CDDP:DOX = 4:1), SaOS-2 and MNNG/HOS cells were also less sensitive to combination chemotherapy than HOS and 143B cells. In contrast, OBP-301 suppressed the viability of SaOS-2 and HOS cells more efficiently as compared to MNNG/HOS and 143B cells. These results indicate that SaOS-2 and MNNG/HOS cells are relatively resistant to chemotherapeutic agents in human osteosarcoma cells.

### Chemosensitizing effect of OBP-301 in human osteosarcoma cells

We have recently shown the synergistic chemosensitizing effect of OBP-301 in human epithelial malignant tumor cells^18^. To investigate the chemosensitizing effect of OBP-301 in human osteosarcoma cells, we treated human osteosarcoma cells with chemotherapeutic agents and OBP-301 in combination therapy to achieve a synergistic effect. The most suitable condition for combination therapy was treatment with the chemotherapeutic agents for 24 hours following OBP-301 infection for 48 hours (Suppl. Fig. 1). When cells were treated with OBP-301 and chemotherapeutic agents (CDDP:DOX = 4:1) by the most suitable combination protocol, cell viability was decreased in a dose-dependent manner (Fig. 1b). The calculation of the combination index demonstrated the synergistic antitumor effect of combination therapy in all 4 human osteosarcoma cell lines (Fig. 1c). In contrast, administration of chemotherapeutic agents did not affect the replication efficacy of OBP-301 in SAOS-2 and MNNG/HOS cells (Suppl. Fig. 2). These results suggest that OBP-301 is a potent chemosensitizer in human osteosarcoma cells.

### Enhancement of chemotherapy-induced apoptosis and DNA damage by OBP-301

**Figure 2:**
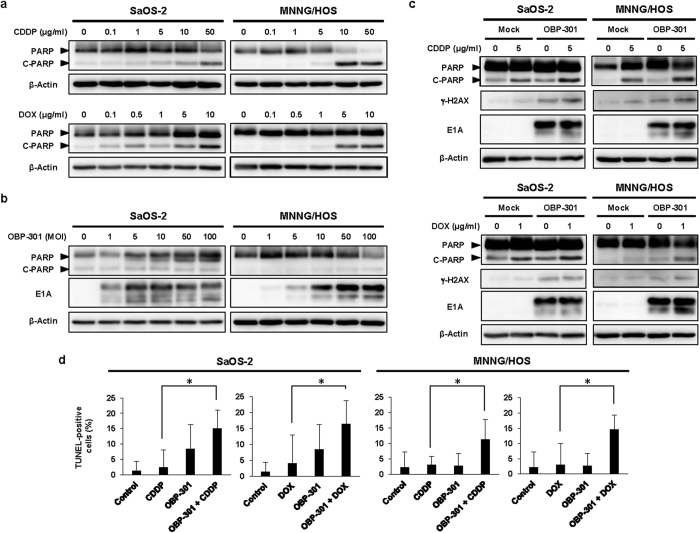
OBP-301 enhances chemotherapy-induced apoptosis and DNA damage. Western blot analysis was performed under the same experimental conditions. (**a**) Expression of PARP and cleaved PARP (C-PARP) proteins in SaOS-2 and MNNG/HOS cells treated with chemotherapeutic agents (CDDP, DOX) at the indicated doses. (**b**) Expression of PARP, C-PARP and adenoviral E1A proteins in SaOS-2 and MNNG/HOS cells treated with OBP-301 at the indicated MOIs. (**c**) Expression of PARP, C-PARP, γ-H2AX, and adenoviral E1A proteins in SaOS-2 and MNNG/HOS cells treated with OBP-301 and chemotherapeutic agents (CDDP, DOX). β-actin was used as a loading control. (**d**) The percentage of apoptotic population was analyzed by TUNEL assay in SaOS-2 and MNNG/HOS cells after treatment with control, CDDP, DOX, OBP-301, or the combination of OBP-301 and chemotherapeutic agents. Cells were infected with OBP-301 at doses of 10 or 50 MOI, respectively. Two days after OBP-301 infection, cells were treated with cisplatin (CDDP) or doxorubicin (DOX) at 5 or 1 μg/ml, respectively, for 24 hours. Data are expressed as mean values ± SD (n = 5). Statistical significance (*) was defined as *P* < 0.05.

**Figure 3:**
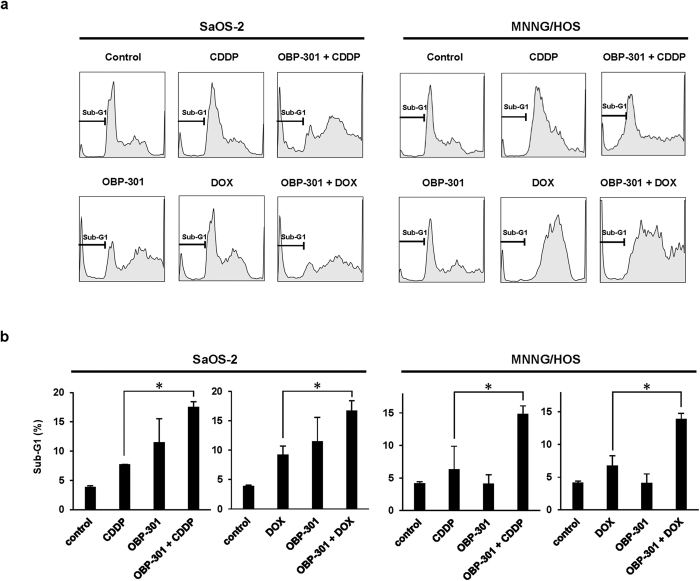
OBP-301-mediated enhancement of apoptosis in combination with chemotherapy. (**a**) The cell-cycle state was analyzed by flow cytometry in SaOS-2 and MNNG/HOS cells stained with propidium iodide after treatment with mock (control), CDDP, DOX, OBP-301, or the combination of OBP-301 and chemotherapeutic agents (CDDP, DOX). SaOS-2 and MNNG/HOS cells were infected with OBP-301 at doses of 10 or 50 MOI, respectively. Two days after OBP-301 infection, cells were treated with cisplatin (CDDP) or doxorubicin (DOX) at 5 or 1 μg/ml, respectively, for 24 hours. Representative cell-cycle data are shown. (**b**) The percentage of sub-G1 population was analyzed using flow cytometry in SaOS-2 and MNNG/HOS cells after treatment with control, CDDP, DOX, OBP-301, or the combination of OBP-301 and chemotherapeutic agents. Data are expressed as mean values ± SD (n = 3). Statistical significance (*) was defined as *P* < 0.05.

To evaluate the underlying mechanism in the OBP-301-mediated enhancement of chemosensitivity, we assessed whether OBP-301 enhances chemotherapy-mediated apoptosis induction and DNA damage. After chemotherapy-resistant SaOS-2 and MNNG/HOS cells were treated with chemotherapy and/or OBP-301, apoptosis induction and DNA damage status were assessed by western blot and flow cytometry. Western blot analysis showed that CDDP and DOX induced apoptosis, which was confirmed by the increased expression of cleaved poly(ADP-ribose) polymerase (PARP), in a dose-dependent manner (Fig. 2a). In contrast, OBP-301 slightly induced the expression of cleaved PARP in MNNG/HOS cells, but not SaOS-2 cells (Fig. 2b). When combined with chemotherapy, OBP-301 enhanced the chemotherapy-induced cleaved PARP expression in SaOS-2 and MNNG/HOS cells (Fig. 2c). Compared with enhancement of cleaved PARP expression, we could not detect the expression of different apoptosis indicator cleaved caspase-3 (data not shown). In contrast, the expression of DNA damage biomarker γ-H2AX was increased in combination therapy compared to monotherapy (Fig. 2c). TUNEL assay demonstrated that the percentage of TUNEL-positive cells was significantly increased in combination therapy compared to monotherapy (Fig. 2d and Suppl. Fig. 3). Moreover, flow cytometric analysis also revealed that OBP-301 significantly increased the percentage of the sub-G1 population, which is a fraction of apoptotic cells, in the chemotherapy-treated SaOS-2 and MNNG/HOS cells (Fig. 3). These results indicate that OBP-301 mediates its chemosensitizing effect through the enhancement of chemotherapy-induced apoptosis and DNA damage.

### Downregulation of anti-apoptotic MCL1 is a critical factor in enhancing chemotherapy-induced apoptosis by OBP-301

**Figure 4:**
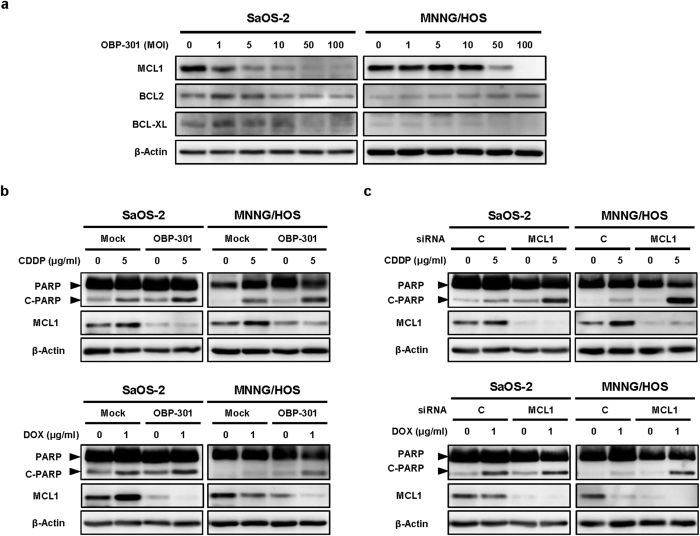
OBP-301-mediated MCL1 suppression enhances chemotherapy-induced apoptosis. Western blot analysis was performed under the same experimental conditions. (**a**) Expression of the anti-apoptotic BCL2 family proteins (MCL1, BCL2, BCL-XL) in SaOS-2 and MNNG/HOS cells infected with OBP-301 at the indicated MOIs for 72 hours. (**b**) Expression of PARP, cleaved PARP (C-PARP) and MCL1 proteins in SaOS-2 and MNNG/HOS cells treated with OBP-301 and chemotherapeutic agents (CDDP, DOX). Cells were infected with OBP-301 at doses of 10 or 50 MOI, respectively. Two days after OBP-301 infection, cells were treated with cisplatin (CDDP) or doxorubicin (DOX) at 5 or 1 μg/ml, respectively, for 24 hours. (**c**) Expression of PARP, C-PARP and MCL1 proteins in SaOS-2 and MNNG/HOS cells treated with chemotherapeutic agents (CDDP, DOX) and MCL1 siRNA or control siRNA. Cells were treated with 10 nmol/L MCL1 siRNA or control siRNA. Two days after siRNA treatment, cells were treated with CDDP or DOX at 5 or 1 μg/ml, respectively, for 24 hours. β-actin was used as a loading control.

Anti-apoptotic BCL2 family proteins, such as BCL2, MCL1, and BCL-XL, play a critical role in the resistance to chemotherapeutic agents^19^. To clarify the molecular mechanism underlying the chemosensitizing effect of OBP-301, we assessed whether OBP-301 affects the expression of anti-apoptotic BCL2 family proteins in chemotherapy-resistant SaOS-2 and MNNG/HOS cells. Western blot analysis showed that OBP-301 dose-dependently downregulated the anti-apoptotic MCL1 protein that was highly expressed in SaOS-2 and MNNG/HOS cells (Fig. 4a). The expression of other BCL2 family proteins BCL2 and BCL-XL was also downregulated after OBP-301 infection in SaOS-2 cells, but OBP-301 slightly increased BCL2 expression and BCL-XL expression was very low in MNNG/HOS cells. Moreover, when combined with chemotherapy, OBP-301 induced MCL1 suppression in consistent with enhancement of chemotherapy-induced apoptosis in SaOS-2 and MNNG/HOS cells (Fig. 4b). These results suggest that OBP-301 enhances chemotherapy-induced apoptosis via MCL1 suppression.

To confirm the role of MCL1 suppression in the OBP-301-mediated enhancement of chemotherapy-induced apoptosis, we further assessed the effect of MCL1 knockdown by RNA interference on the chemotherapy-induced apoptosis. When combined with chemotherapeutic agents, MCL1 siRNA efficiently suppressed MCL1 expression and enhanced the chemotherapy-induced apoptosis as well as OBP-301 in SaOS-2 and MNNG/HOS cells (Fig. 4c). These results suggest that MCL1 suppression plays a crucial role in the OBP-301-mediated enhancement of chemotherapy-induced apoptosis.

### OBP-301-mediated microRNA-29 upregulation suppresses MCL1 expression through E2F1 activation

**Figure 5:**
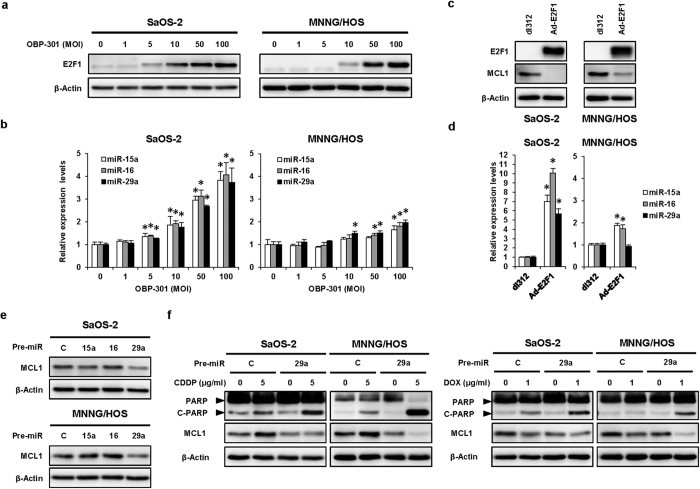
OBP-301-mediated microRNA-29 up-regulation suppresses MCL1 expression and enhances chemotherapy-induced apoptosis. Western blot analysis was performed under the same experimental conditions. (**a**) Expression of E2F1 protein in SaOS-2 and MNNG/HOS cells infected with OBP-301 at the indicated MOIs for 72 hours. (**b**) Expression of miR-15a, miR-16, and miR-29a in SaOS-2 and MNNG/HOS cells infected with OBP-301 at the indicated MOIs for 72 hours. The values of miR-15a, miR-16, and miR-29a at 0 MOI were set at 1, and the relative levels of miR-15a, miR-16, miR-29a at the indicated MOIs were plotted as fold induction. Data are expressed as mean values ± SD (n = 3). Statistical significance (*) was defined as *P* < 0.05. (**c**) Expression of E2F1 and MCL1 proteins in SaOS-2 and MNNG/HOS cells infected with Ad-E2F1 at 100 and 1000 MOIs, respectively, for 48 hours. (**d**) Expression of miR-15a, miR-16, and miR-29a in SaOS-2 and MNNG/HOS cells infected with Ad-E2F1 at 100 and 1000 MOIs, respectively, for 48 hours. (**e**) Expression of MCL1 protein in SaOS-2 and MNNG/HOS cells treated with 10 nmol/L Pre-miR-15a (15a), Pre-miR-16 (16), Pre-miR-29a (29a), or control Pre-miRNA (C) for 72 hours. (**f**) Expression of PARP, C-PARP and MCL1 proteins in SaOS-2 and MNNG/HOS cells treated with chemotherapeutic agents (CDDP, DOX) and control Pre-miRNA (C) or miR-29a (29a). Cells were treated with 10 nmol/L Pre-miR-29a or control Pre-miRNA. Two days after miRNA treatment, cells were treated with cisplatin (CDDP) or doxorubicin (DOX) at 5 or 1 μg/ml, respectively, for 24 hours. β-actin was used as a loading control.

We recently revealed that OBP-301^20^ and tumor suppressor p53-expressing OBP-301 (OBP-702)^21^ increase the expression of cellular microRNAs (miRNAs) miR-7 and miR-93/106b, respectively, via activation of transcription factor E2F1 in human cancer cells. miR-15, miR-16, and miR-29 suppress MCL1 expression in human malignant tumor cells^22^. Moreover, a recent report has suggested that miR-16 and miR-29 are downregulated and miR-15 is associated with chemosensitivity in human osteosarcoma cells^23^. To investigate the underlying mechanism of OBP-301-mediated MCL1 suppression, we determined whether OBP-301 upregulates MCL1-targeted miRNAs (miR-15, miR-16, miR-29) via E2F1 activation in human osteosarcoma cells. OBP-301 dose-dependently upregulated the expression of E2F1 and MCL1-targeted miRNAs (miR-15a, miR-16, miR-29a) in SaOS-2 and MNNG/HOS cells (Fig. 5a,b). In contrast, when infected with E2F1-expressing Ad-E2F1 or control E1A-deleted adenovirus dl312, Ad-E2F1 increased E2F1 expression and inversely decreased MCL1 expression in SaOS-2 and MNNG/HOS cells (Fig. 5c). Moreover, Ad-E2F1 significantly increased the expression of miR-15a, miR-16, and miR-29a in SaOS-2 cells, although MNNG/HOS cells showed increased expression of miR-15a and miR-16, but not miR-29a, after Ad-E2F1 infection (Fig. 5d). These results suggest that OBP-301 induces MCL1-targeted miRNAs via E2F1 activation in human osteosarcoma cells.

To evaluate the effect of MCL1-targeted miRNAs in the chemosensitivity of human osteosarcoma cells, we introduced exogenous miR-15a, miR-16, miR-29a or control miRNA into SaOS-2 and MNNG/HOS cells. Only miR-29a efficiently suppressed MCL1 expression in SaOS-2 and MNNG/HOS cells (Fig. 5e). Moreover, miR-29a enhanced the chemotherapy-induced apoptosis in SaOS-2 and MNNG/HOS cells (Fig. 5f). These results suggest that OBP-301 enhances chemotherapy-induced apoptosis through miR-29-mediated MCL1 suppression in human osteosarcoma cells.

### Suppression of MNNG/HOS tumor growth by the combined treatment of chemotherapeutic agents and OBP-301

**Figure 6:**
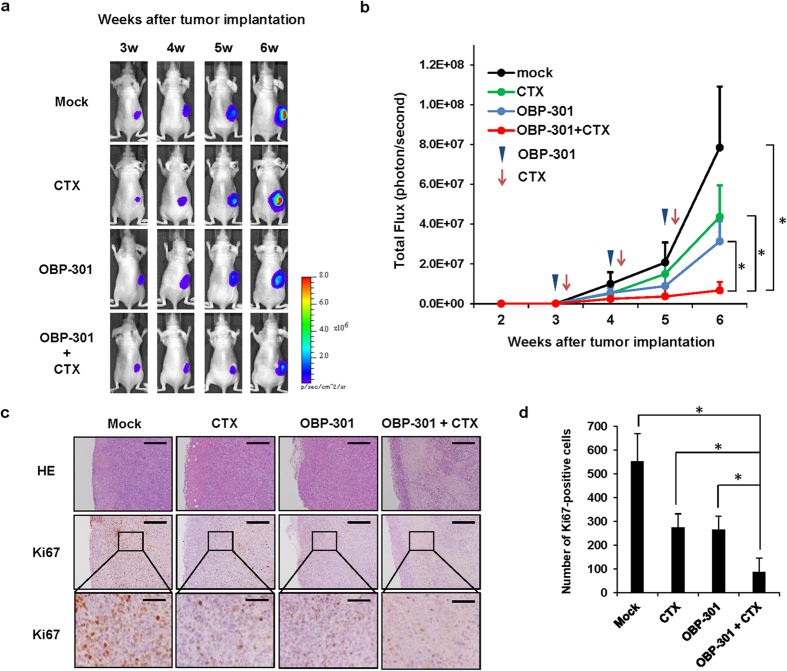
Enhancement of chemotherapy-mediated antitumor effect in combination with OBP-301 in subcutaneous MNNG/HOS osteosarcoma xenograft model. Athymic nude mice were inoculated subcutaneously with MNNG/HOS-Luc cells (5 × 10^6^ cells/site). OBP-301 (5 × 10^7^ PFU) was injected into the tumor at 3, 4, and 5 weeks after tumor inoculation. Cisplatin (4 mg/kg body weight) and doxorubicin (2 mg/kg body weight) were intraperitoneally injected 2 days after OBP-301 injection in chemotherapy (CTX) treatment. **(a)** Representative photographs of tumor-bearing mice treated with PBS (mock), CTX, OBP-301, or OBP-301 and CTX. **(b)** The luminescence in tumor tissues was analyzed using the IVIS system at 2, 3, 4, 5, and 6 weeks after tumor inoculation. Data are expressed as mean values ± SD (n = 4). (**c**) histologic analysis of the MNNG/HOS tumors. Tumor tissues were obtained at 6 weeks after tumor inoculation. Paraffin-embedded sections of MNNG/HOS tumors were stained with hematoxylin and eosin solutions or anti-Ki67 antibody. Upper and middle images are low magnification in HE and Ki67 staining, respectively. Lower images are high magnification of the area outlined by a white square in Ki67 staining. Upper and middle scale bars, 200 μm. Lower scale bars, 50 μm. (**d**) the number of Ki67-positive cells in tumor tissue was calculated using ImageJ software. Statistical significance (*) was defined as *P* < 0.05.

Finally, to assess the *in vivo* antitumor effect of combination therapy with chemotherapy and OBP-301, we used a subcutaneous MNNG/HOS xenograft tumor model because only MNNG/HOS cells have tumorigenic ability^24^. OBP-301 or PBS (mock) was injected into the tumors every week, whereas cisplatin and doxorubicin were intraperitoneally injected 2 days after OBP-301 injection for 3 cycles. The combination of chemotherapeutic agents and OBP-301 significantly suppressed tumor growth when compared to mock or monotherapy with chemotherapeutic agents or OBP-301 (Fig. 6a,b). On histopathological analysis, there were large necrotic areas with decreased Ki-67 expression in the combination therapy-treated tumors as compared to mock-treated, OBP-301- or chemotherapy-treated tumors (Fig. 6c). Combination therapy with OBP-301 and chemotherapy significantly decreased the number of Ki67-positive proliferating cells within tumor tissues compared to mock or monotherapy (Fig. 6d). These results suggest that OBP-301 has the therapeutic potential to enhance the chemotherapy-mediated antitumor effect in human osteosarcoma tumors.

## Discussion

The exploration of the molecular mechanism underlying chemoresistance and the development of novel strategies for enhancing chemosensitivity are pivotal approaches to improve the clinical outcome of osteosarcoma patients. The present study demonstrated that a tumor-specific replication-competent oncolytic adenovirus OBP-301 induced miR-29 upregulation via E2F1 activation, which resulted in suppression of anti-apoptotic BCL2 family protein MCL1 in chemotherapy-resistant human osteosarcoma cells. OBP-301 synergistically enhanced the chemosensitivity in human osteosarcoma cells by promoting chemotherapy-induced apoptosis and DNA damage. Although oncolytic adenoviruses hTERT-Ad^25^, SG511^26^, and OBP-301^27^,^28^,^29^,^30^ showed chemosensitizing effects in epithelial types of malignant tumor cells, the precise molecular mechanism underlying the adenovirus-mediated chemosensitization remained unclear, especially in human osteosarcoma cells. Virally triggered MCL1 depletion may be a novel, promising strategy to overcome chemoresistance in human osteosarcomas. Moreover, activation of miRNA networks targeting MCL1 is essential for this clinically relevant chemovirotherapy.

MCL1 is overexpressed in various types of human tumor cells^31^; thus, MCL1 has emerged as a potential therapeutic target. Some reports have suggested that MCL1 suppression by MCL1 siRNA and antisense oligonucleotides enhance the antitumor effects of chemotherapy in human cancers including sarcomas^7^, hepatocellular carcinomas^32^, pancreatic cancers^33^ and gastric cancers^34^. MCL1-targeted drugs, such as small molecule inhibitors, have recently been developed to improve the chemotherapy-mediated antitumor effect in human cancer cells^19^. However, MCL1 suppression also induces apoptosis in normal hematopoietic cells, such as B and T lymphocytes^35^, polymorphonuclear leukocytes^36^, macrophages^37^ and hematopoietic stem cells^38^. To avoid the cytotoxicity of MCL1 suppression in normal hematopoietic cells, OBP-301-mediated tumor-specific MCL1 ablation might be clinically beneficial.

Regarding the molecular mechanism underlying the oncolytic adenovirus-mediated MCL1 suppression, we demonstrated that OBP-301 upregulated MCL1-targeted miRNAs, such as miR-15, miR-16 and miR-29, and miR-29 overexpression efficiently suppressed MCL1 expression in human osteosarcoma cells. Recent reports have shown that MCL1 is a direct target of miR-29a, which binds to 3′-UTR of MCL1, in human tumor cells including SaOS-2 cells^39^,^40^,^41^. Regarding the function of miR-29 in tumor development, the miR-29 family is frequently downregulated in human cancers^42^ including osteosarcomas^43^, suggesting the tumor-suppressive role of the miR-29 family. In fact, our data demonstrated that administration of a miR-29 mimic enhanced the chemotherapy-induced apoptosis like MCL1 siRNA. Moreover, miR-29 negatively regulates the expression of stemness-related markers, such as Oct3/4, Sox2 and Nanog, and subsequently suppresses the proliferation, sphere formation and chemoresistance of human osteosarcoma stem cells^43^. Therefore, OBP-301-mediated miR-29 activation may have broad therapeutic potential in addition to chemosensitization through MCL1 depletion. However, miR-15a, miR-16 and miR-29a may not be the only MCL1-targeted miRNAs because we cannot exclude the possible involvement of other miRNAs in regulating MCL1 expression. Comprehensive miRNA expression profiling by using either a miRNA microarray or a miRNA PCR array, therefore, will be necessary to clarify this issue in the future study.

OBP-301 treatment increased the level of miR-29a in SaOS-2 and MNNG/HOS cells. However, E1A-deficient Ad-E2F1-induced E2F1 overexpression was not sufficient to increase the level of miR-29a in MNNG/HOS cells. Previous report has suggested that adenoviral E1A accumulation is necessary to suppress MCL1 expression in adenovirus-infected cells^44^. Adenovirus induces E2F1 expression via E1A-mediated inactivation of E2F1-suppressive retinoblastoma (Rb) protein and activation of free E2F1^45^. Since SaOS-2 cells lack Rb protein, but HOS cells, which are parental cells of MNNG/HOS cells, have Rb protein^46^, inactivation of E2F1-suppressive Rb protein in addition to E2F1 overexpression may be necessary to increase the level of miR-29a in MNNG/HOS cells.

We and our collaborators recently confirmed the antitumor effect of OBP-301 in human osteosarcoma cells as a monotherapy^47^,^48^. A Phase I clinical trial in the United States showed the safety and feasibility of intratumoral injection of OBP-301 in patients with various types of advanced solid tumors including sarcomas^49^. Although we have recently confirmed more profound antitumor effect of p53-expressing OBP-702 compared to OBP-301 against human osteosarcoma cells^21^, the safety and feasibility of OBP-702 treatment in cancer patients remains to be elucidated. For future clinical application, we focused on the therapeutic potential of OBP-301 in a multimodal antitumor strategy with chemotherapy in this study. Recent evidence has shown not only the antitumor effect, but also the chemosensitizing effect of oncolytic adenoviruses in human cancer cells^18^,^50^. For example, we confirmed that OBP-301 enhances the antitumor effect of chemotherapeutic agents, such as docetaxel^27^, gemcitabine^28^, CDDP 29,30, and paclitaxel^30^, in human cancer cells with telomerase activities. In this study, we demonstrated the potential of OBP-301 as a chemosensitizing reagent against osteosarcoma cells in *in vitro* and *in vivo* experiments. Chemosensitizing effect of OBP-301 was more striking in *in vivo* experiment compared to *in vitro* experiment. The difference between *in viro* and *in vivo* chemosensitizing effect of OBP-301 may be in part due to different frequency of treatment, one time (*in vitro*) and three times (*in vivo*). Short-term *in vivo* experiment may be necessary to precisely confirm the apoptosis induction status within tumor tissues as well as *in vitro* experiment. In contrast, quiescent tumor cells are predominantly more resistant to chemotherapeutic agents than proliferating tumor cells within tumor tissues^51^. We recently reported that OBP-301 suppressed cell viability in chemoradiotherapy-resistant quiescent human gastric cancer stem cells through induction of cell cycle reentry^52^. In this report, OBP-301 also enhanced the chemotherapy-mediated antitumor effect against dormant human gastric cancer stem cell-driven tumors. OBP-301-mediated modulation of the cell cycle status may also attenuate the chemoresistance of dormant human osteosarcoma cells within tumor tissues.

Our findings suggest that miR-29-mediated suppression of anti-apoptotic factor MCL1 was a critical factor for the enhancement of chemosensitivity in human osteosarcoma cells. Thus, virus-mediated telomerase-specific targeting of MCL-1 expression offers a promising strategy to improve the clinical benefits of conventional chemotherapy in osteosarcoma patients. Further clinical studies are warranted to investigate the tolerability and efficacy of this multimodal combination therapy.

## Materials and Methods

### Cell lines

Four human osteosarcoma cell lines were used in the study. HOS and SaOS-2 cells were kindly provided by Dr. Satoru Kyo (Shimane University, Izumo, Japan). MNNG/HOS cells were purchased from DS Pharma Biomedical, and 143B cells were obtained from the American Type Culture Collection (Manassas, VA, USA). HOS and SaOS-2 cells were maintained in Dulbecco’s Modified Eagle’s Medium. MNNG/HOS cells were maintained in Eagle’s Minimum Essential Medium containing 1% nonessential amino acids. 143B cells were maintained in Eagle’s Minimum Essential Medium containing 0.015 mg/ml 5-bromo-2′-deoxyuridine. MNNG/HOS cells transfected with the firefly luciferase plasmid vector (MNNG/HOS-Luc) were maintained in medium containing 0.2 mg/ml Geneticin (G418; Invitrogen, Carlsbad, CA, USA). All media were supplemented with 10% fetal bovine serum (St. Louis, MO, USA), 100 U/ml penicillin and 100 mg/ml streptomycin. The cells were routinely maintained at 37 °C in a humidified atmosphere with 5% CO_2_.

### Recombinant adenoviruses

Construction and characterization of the recombinant telomerase-specific replication-competent adenovirus vector OBP-301 (telomelysin) was previously reported^16^,^17^. Replication-deficient adenovirus vector expressing E2F1 (Ad-E2F1) was used to induce E2F1 expression in infected cells, as previously reported^53^. E1A-deleted adenovirus vector (dl312) was used as a control vector. OBP-301, Ad-E2F1 and dl312 were purified by using CsCl step gradient ultracentrifugation followed by CsCl linear gradient ultracentrifugation. This study was approved by the Recombinant DNA Experiment Safety Committee and carried out in accordance with the approved protocol (Approved ID: 12015).

### Cell viability assay

Cells were seeded on 96-well plates at a density of 1 × 10^3^ cells/well 24 hours before administration of chemotherapeutic drugs or viral infection. In monotherapy, cells were treated with CDDP or DOX at a concentration of 0, 0.1, 1, 5, or 10 μg/ml for 24 hours or infected with OBP-301 at multiplicity of infections (MOIs) of 0, 1, 5, 10, 50, or 100 plaque forming units (PFUs)/cell for 72 hours. In combined chemotherapy, cells were treated with chemotherapeutic agents at the ratio used clinically (CDDP:DOX = 4:1). Moreover, in combination therapy with chemotherapy and OBP-301, two days after viral infection with OBP-301 at the indicated MOIs, cells were further treated with chemotherapy (CDDP:DOX = 4:1) for 24 hours. Cell viability was determined using the Cell Proliferation Kit II (Roche Molecular Biochemicals, Indianapolis, IN, USA) according to the manufacturer’s protocol. The combination index was calculated with CalcuSyn software (BioSoft, Inc., Cambridge, UK), and the computation of the combination index was based on the methods of Chou and Talalay^54^.

### Western blot analysis

SaOS-2 and MNNG/HOS cells seeded in a 100-mm dish at a density of 2 × 10^5^ cells/dish were infected with OBP-301, Ad-E2F1, or dl312 at the indicated MOIs for 72 hours. Cells were treated with CDDP or DOX at the indicated doses for 24 hours. Cells were transfected with 10 nM MCL1 siRNA, control siRNA, Pre-miR-15a, Pre-miR-16, Pre-miR-29a, or control Pre-miRNA (Applied Biosystems, Foster City, CA, USA) 48 hours before chemotherapy treatment and treated with CDDP or DOX at the indicated doses for 24 hours. Whole cell lysates were prepared in a lysis buffer (50 mM Tris-HCl (pH 7.4), 150 mM NaCl, 1% Triton X-100) containing a protease inhibitor cocktail (Complete Mini; Roche Applied Science, Mannheim, Germany). Proteins were electrophoresed on 8–15% sodium dodecyl sulfate polyacrylamide gels and transferred to polyvinylidene difluoride membranes (Hybond-P; GE Health Care, Buckinghamshire, UK). The membranes were blocked with Blocking-One (Nacalai Tesque, Kyoto, Japan) at room temperature for 30 minutes. The primary antibodies used were: mouse anti-Ad5 E1A monoclonal antibody (mAb) (BD PharMingen, Franklin Lakes, NJ, USA); rabbit anti-PARP polyclonal antibody (pAb), mouse anti-γ-H2AX mAb (Upstate Biotechnology Inc., Temecula, CA, USA), rabbit anti-MCL1 mAb, rabbit anti-BCL-2 mAb, rabbit anti-BCL-XL mAb, and rabbit anti-E2F1 mAb (Cell Signaling Technology, Danvers, MA, USA); and mouse anti-β-actin mAb (Sigma-Aldrich). The secondary antibodies used were: horseradish peroxidase-conjugated antibodies against rabbit IgG (GE Healthcare), mouse IgG (GE Healthcare) or goat IgG (Chemicon International Inc., Temecula, CA, USA). Immunoreactive bands on the blots were visualized using enhanced chemiluminescence substrates (ECL Plus; GE Healthcare).

### DNA fragmentation analysis

SaOS-2 and MNNG/HOS cells seeded in a 4 well glass plate at a density of 5 × 10^3^ cells/well were treated with or without CDDP (5 μg/ml), DOX (1 μg/ml), and/or OBP-301 (10 and 50 MOIs, respectively). In monotherapy, cells were treated with chemotherapy for 24 hours or infected with OBP-301 for 72 hours. In combination therapy, two days after viral infection with OBP-301, cells were further treated with chemotherapy for 24 hours. Cells were fixed in 1% paraformaldehyde in PBS at 4 °C for 10 minutes. Cells were stained by nick end-labelling method to detect the fragmented DNA, and Hoechst 33342 (Thermo Fisher Scientific Inc., Rockford, IL, USA) to observe the nuclear morphology of the cells. Briefly, cells were incubated with TdT reaction solution (400 U terminal transferase and 5 nmol Fluorescen-12-2′-deoxy-uridine-5′-triphosphate in 1.2 ml TUNEL Dilution Buffer (Roche Diagnostics, Mannheim, Germany)). The cells were then stained by Hoechst 33342 (20 μM) for 5 minutes at room temperature. The photographs of immunostained sections were obtained by fluorescent microscopy. The percentage of immunoreactive cells for TUNEL was calculated in five randomly selected fields in each group.

### Flow cytometric analysis

To evaluate the sub-G1 population, which is an apoptosis indicator, the cell cycle state was analyzed by flow cytometry, as previously reported^21^. SaOS-2 and MNNG/HOS cells seeded in a 100-mm dish at a density of 1 × 10^6^ cells/dish were treated with or without CDDP (5 μg/ml), DOX (1 μg/ml), and/or OBP-301 (10 and 50 MOIs, respectively). In monotherapy, cells were treated with chemotherapy for 24 hours or infected with OBP-301 for 72 hours. In combination therapy, two days after viral infection with OBP-301, cells were further treated with chemotherapy for 24 hours. Cells were trypsinized and resuspended in original supernatant to ensure that attached and nonattached cells were analyzed. Cells stained with propidium iodide were analyzed using a FACS array (BD Biosciences, Franklin Lakes, NJ, USA).

### Quantitative real-time reverse transcription-PCR analysis

To evaluate the expression of miR-15a, miR-16, and miR-29a in tumor cells after OBP-301 infection, SaOS-2 and MNNG/HOS cells were seeded on 6-well plates at a density of 2 × 10^5^ cells/well and 24 hours later infected with OBP-301 at MOIs of 0, 1, 5, 10, 50, or 100 PFU/cell. Three days after virus infection, total RNA was extracted from cells using a miRNeasy Mini Kit (Qiagen, Valencia, CA, USA). After synthesis of cDNA from 10 ng of total RNA using the TaqMan MicroRNA Reverse Transcription Kit (Applied Biosystems), the expression of miR-15a, miR-16, or miR-29a was determined by quantitative real-time RT-PCR (qRT-PCR) using the Applied Biosystems StepOnePlus^TM^ real-time PCR system. The expression levels of miR-15a, miR-16, and miR-29a were defined from the threshold cycle (Ct), and relative expression levels were calculated using the 2^−ΔΔCt^ method after normalization with reference to the expression of U6 small nuclear RNA.

### *In vivo* MNNG/HOS xenograft tumor model

Animal experimental protocols were approved by the Ethics Review Committee for Animal Experimentation of Okayama University School of Medicine (No. OKU-2011062). MNNG/HOS-Luc cells (5 × 10^6^ cells per site) were inoculated into the flanks of 5- to 6-week-old female BALB/c *nu/nu* mice (CLEA Japan, Tokyo, Japan). Palpable tumors developed within 21 days and were permitted to grow to approximately 5 to 6 mm in diameter. At that stage, a 50 μL volume of solution containing OBP-301 (5 × 10^7^ PFU) or PBS was injected into the tumors every week for 3 cycles. Cisplatin (4 mg/kg body weight) and doxorubicin (2 mg/kg body weight) were intraperitoneally injected 2 days after OBP-301 injection for 3 cycles. Four mice were used for each group. To monitor tumor progression, the substrate luciferin (VivoGlo Luciferin; Promega, Madison, WI, USA) was intraperitoneally injected at a dose of 3 mg/kg body weight. Images were collected in the prone position after luciferin injection with the Xenogen IVIS Lumina Imaging System (Caliper Life Sciences, Cheshire, UK), and photons emitted from the flanks were quantified by using Xenogen Living Image Software (Caliper Life Science).

### Histopathologic and immunohistochemical analysis

Tumors were fixed in 10% neutralized formalin and embedded in paraffin blocks. Paraffin-embedded sections (4 μm) were prepared for hematoxylin/eosin (H&E) staining and immunohistochemical examination. Immunostaining with rabbit anti-Ki67 mAb (Abcam, Cambridge, MA, USA) using standard techniques was used to detect proliferating tumor cells within tumor tissues. The photographs of immunostained sections were obtained by light microscopy. The number of immunoreactive cells for Ki67 was calculated in five randomly selected fields in each tumor by using ImageJ software.

### Statistical analysis

Data are expressed as means ± SD. Determination of significant differences was assessed using the Student’s *t*-test. *P* values less than 0.05 were considered to indicate statistical significance.

## Additional Information

**How to cite this article**: Osaki, S. *et al*. Ablation of MCL1 expression by virally induced microRNA-29 reverses chemoresistance in human osteosarcomas. *Sci. Rep.* **6**, 28953; doi: 10.1038/srep28953 (2016).

## Supplementary Material

Supplementary Information
