# Supplementary material for: Ablation of MCL1 expression by virally induced microRNA-29 reverses chemoresistance in human osteosarcomas
Source: Sci Rep. 2016 Jun 30;6:28953. doi: 10.1038/srep28953 (PMC4928055; doi:10.1038/srep28953)
Supplement: Supplementary Information [file srep28953-s1.pdf]

Supplementary Information

Ablation of MCL1 expression by virally induced MicroRNA-29 reverses chemoresistance in human osteosarcomas

Shuhei Osaki[1], Hiroshi Tazawa[2,3], Joe Hasei[1], Yasuaki Yamakawa[1], Toshinori Omori[1],

Kazuhisa Sugiu[1], Tadashi Komatsubara[1], Tomohiro Fujiwara[1,3], Tsuyoshi Sasaki[1],

Toshiyuki Kunisada[1,4], Aki Yoshida[1], Yasuo Urata[5], Shunsuke Kagawa[2],

Toshifumi Ozaki[1] & Toshiyoshi Fujiwara[2]

Departments of [1]Orthopaedic Surgery, [2]Gastroenterological Surgery, and [4]Medical Materials for Musculoskeletal Reconstruction, Okayama University Graduate School of Medicine, Dentistry and Pharmaceutical Sciences, Okayama 700-8558, Japan. [3]Center for Innovative Clinical Medicine, Okayama University Hospital, Okayama 700-8558, Japan. [5]Oncolys BioPharma, Inc., Tokyo 105-0001, Japan

Supplementary Materials and Methods

Supplementary Figure 1

Protocol and combination index in combination therapy
with chemotherapeutic agent and OBP-301.

Supplementary Figure 2

Quantitative measurement of viral DNA replication in MNNG/HOS cells
treated with OBP-301 and chemotherapeutic agents.

Supplementary Figure 3

Induction of apoptotic cells in SaOS-2 and MNNG/HOS cells
treated with OBP-301 and chemotherapeutic agents.

Supplementary Materials and Methods

Cell viability assay

HOS cells, seeded on 96-well plates at a density of 1 x 10^3 cells/well, were infected with OBP-301 at 0, 10, or 100 PFU/cell and/or treated with CDDP at 0, 0.5, 2.5, or 10 μg/ml at the indicated time points. Cell viability was determined using Cell Proliferation Kit II (Roche Molecular Biochemicals) according to the manufacturer's protocol. The combination index was calculated with the CalcuSyn software (BioSoft, Inc.).

Quantitative real-time PCR analysis

To compare the E1A copy number between OBP-301-infected cells with and without chemotherapeutic agents (CDDP, DOX), MNNG/HOS cells, seeded on 6-well plates at a density of 5 x 10^5 cells/well, were infected with OBP-301 at an MOI of 50 PFUs/cell. Then, cells were treated with CDDP (0.5 μg/ml) or DOX (0.1 μg/ml) at 2, 24 or 48 hours after OBP-301 infection. Genomic DNA was extracted from serially diluted viral stocks and tumor cells by using the QIAmp DNA Mini Kit (Qiagen). E1A copy number was also determined using TaqMan real-time PCR systems (Applied Biosystems).

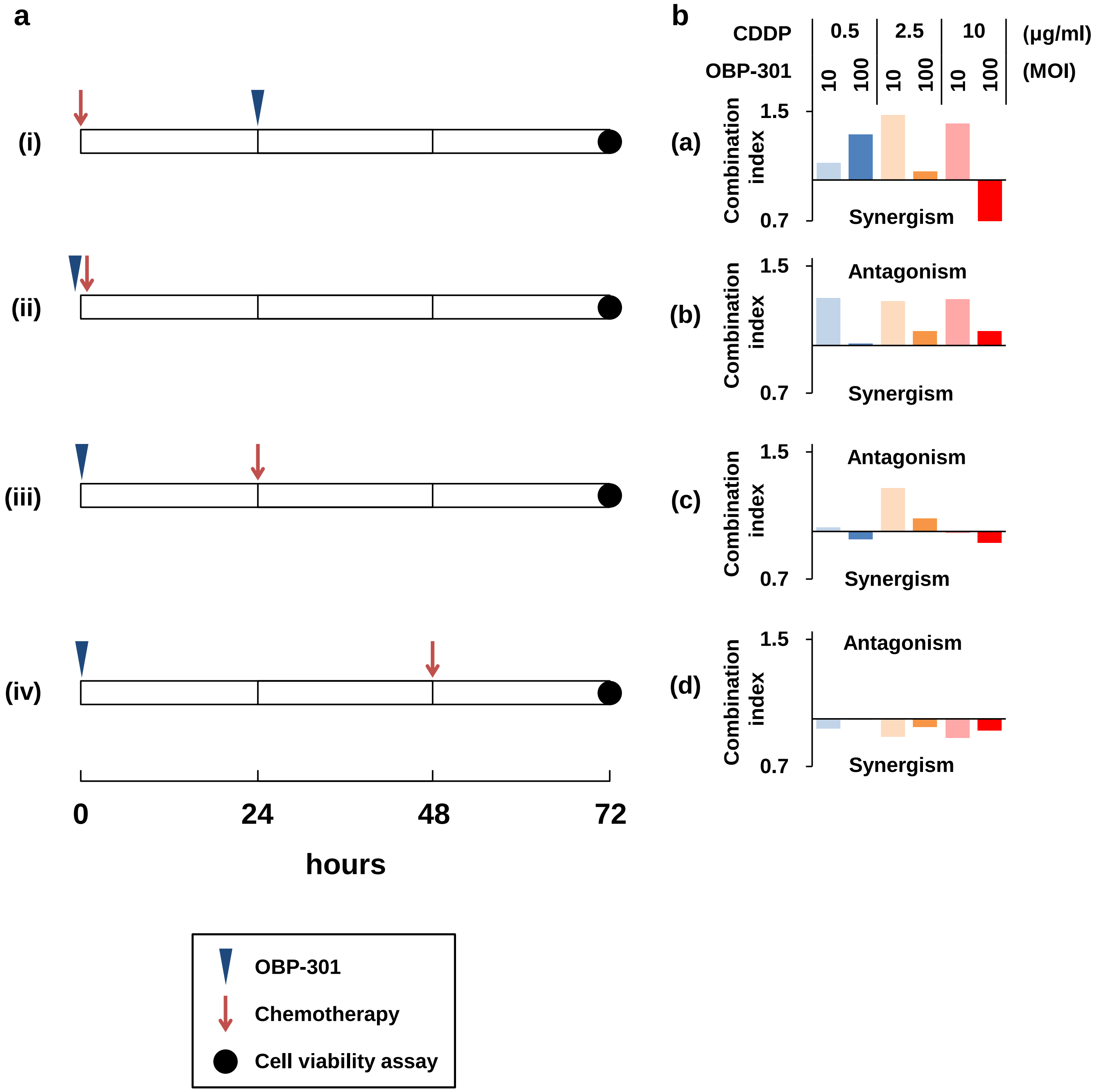

Supplemental Figure 1

Protocol and combination index in combination therapy with chemotherapeutic agent and OBP-301. **(a)** a scheme for protocol of combination therapy with chemotherapy and OBP-301. HOS cells were treated with chemotherapy and OBP-301 at the indicated time points (a, b, c, d). Cell viability assay was performed at 72 hours after first treatment. **(b)** The combination index in combination therapy (i, ii, iii, iv) was calculated with the CalcuSyn software. Synergism and antagonism were defined as interaction indices of < 1 or > 1, respectively.

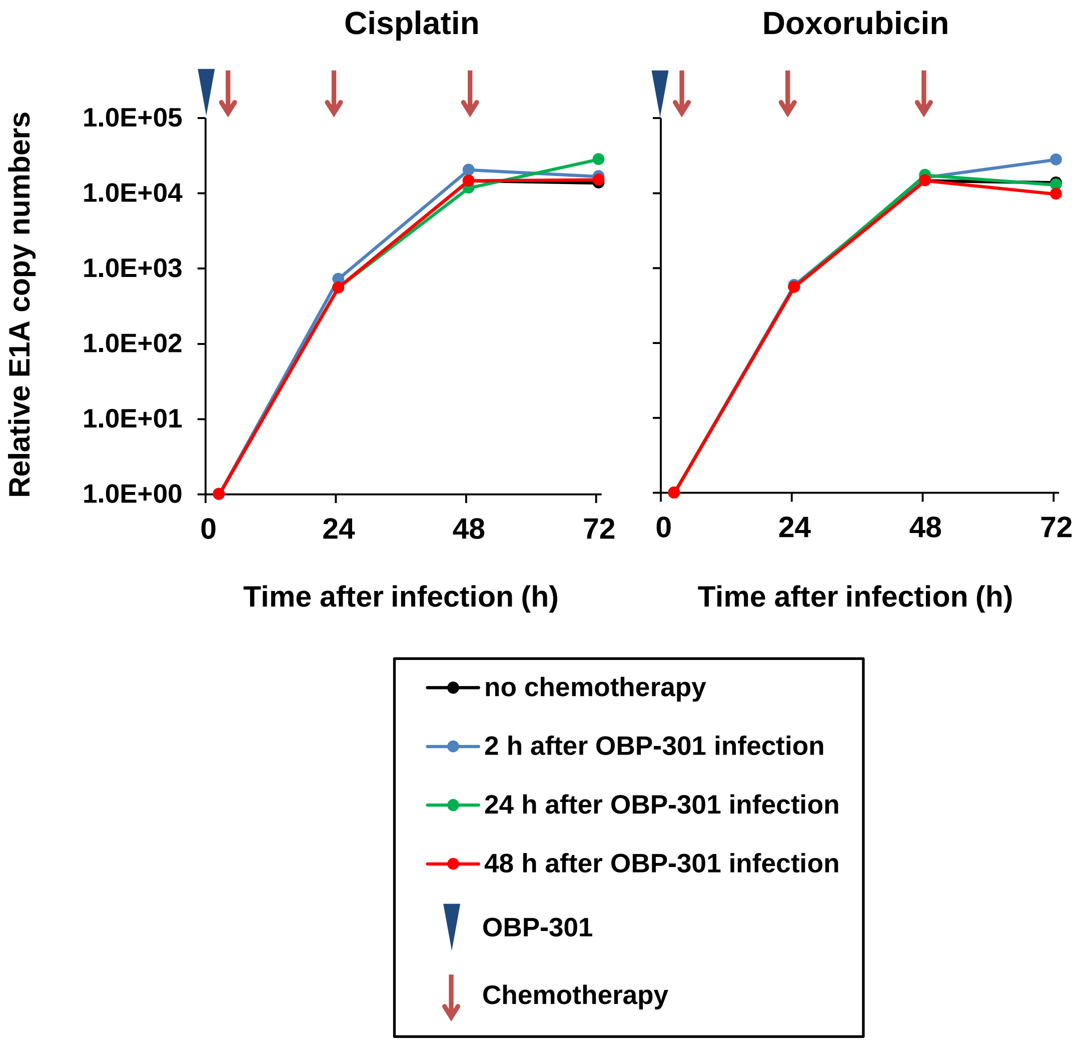

Supplementary Figure 2

Quantitative measurement of viral DNA replication in MNNG/HOS cells treated with OBP-301 and chemotherapeutic agents. The cells were infected with OBP-301 at an MOI of 50 PFUs/cell, and treated with cisplatin (0.5 μg/ml) or doxorubicin (0.1 μg/ml) at 2, 24 or 48 hours after OBP-301 infection. E1A copy number was analyzed over the following 3 days after OBP-301 infection by quantitative real-time PCR. The value of the E1A copy number at 2 hours after OBP-301 infection was set at 1, and relative copy numbers were plotted.

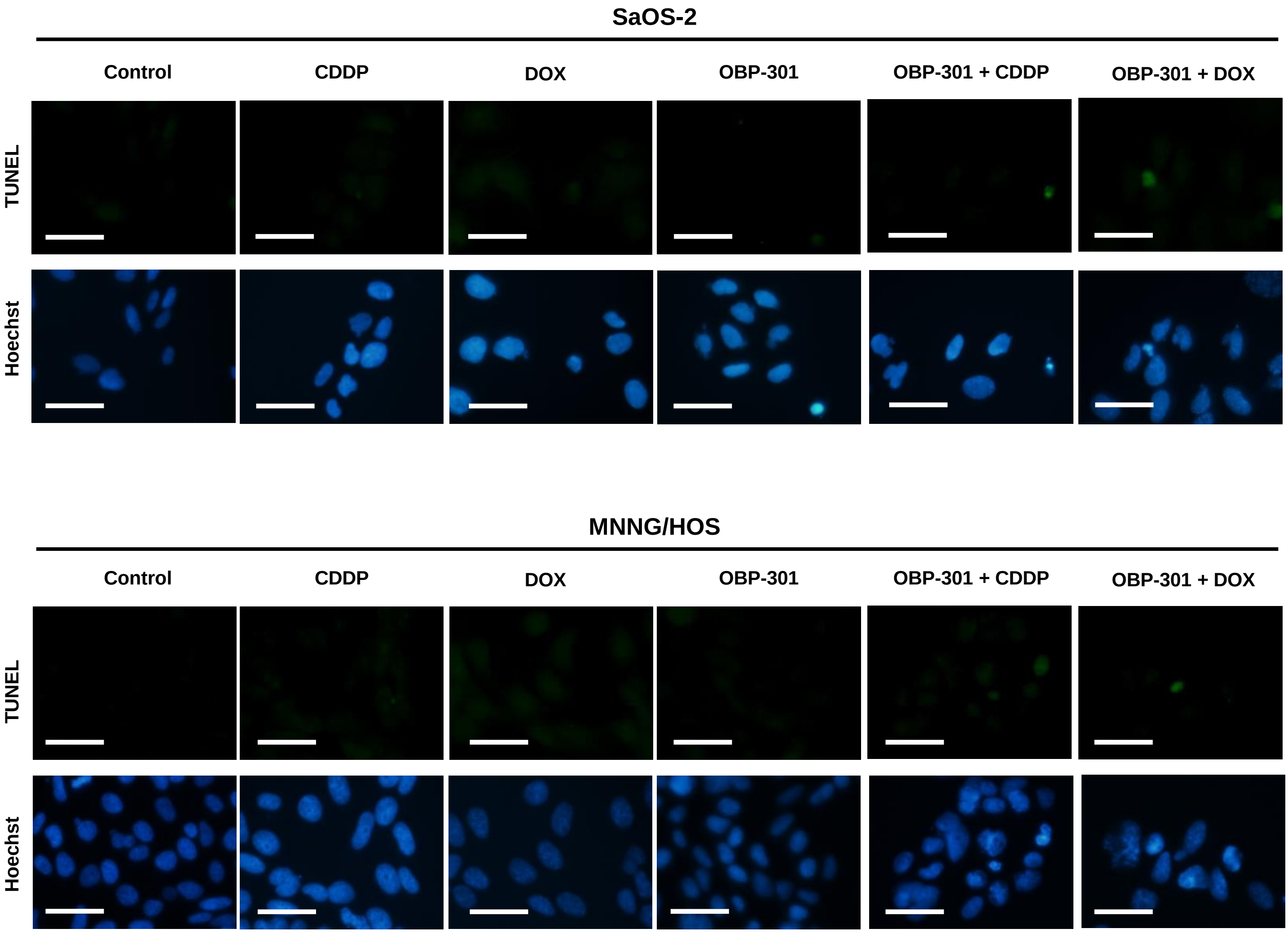

Supplementary Figure 3

Induction of apoptotic cells in SaOS-2 and MNNG/HOS cells treated with OBP-301 and chemotherapeutic agents. SaOS-2 and MNNG/HOS cells were infected with OBP-301 at doses of 10 or 50 MOI, respectively. Two days after OBP-301 infection, cells were treated with cisplatin (CDDP) or doxorubicin (DOX) at 5 or 1 μg/ml, respectively, for 24 hours. The representative photographs for cells stained by TUNEL assay and Hoechst in each group are shown. Scale bars, 50 μm.
